# Phagocytic Superoxide Specifically Damages an Extracytoplasmic Target to Inhibit or Kill *Salmonella*

**DOI:** 10.1371/journal.pone.0004975

**Published:** 2009-03-23

**Authors:** Maureen Craig, James M. Slauch

**Affiliations:** 1 Department of Microbiology, University of Illinois, Urbana, Illinois, United States of America; 2 College of Medicine, University of Illinois, Urbana, Illinois, United States of America; Massachusetts General Hospital, United States of America

## Abstract

**Background:**

The phagocytic oxidative burst is a primary effector of innate immunity that protects against bacterial infection. However, the mechanism by which reactive oxygen species (ROS) kill or inhibit bacteria is not known. It is often assumed that DNA is a primary target of oxidative damage, consistent with known effects of endogenously produced ROS in the bacterial cytoplasm. But most studies fail to distinguish between effects of host derived ROS versus damage caused by endogenous bacterial sources. We took advantage of both the ability of *Salmonella enterica* serovar Typhimurium to survive in macrophages and the genetic tractability of the system to test the hypothesis that phagocytic superoxide damages cytoplasmic targets including DNA.

**Methodology/Principal Findings:**

SodCI is a periplasmic Cu-Zn superoxide dismutase (SOD) that contributes to the survival of *Salmonella* Typhimurium in macrophages. Through competitive virulence assays, we asked if *sodCI* has a genetic interaction with various cytoplasmic systems. We found that SodCI acts independently of cytoplasmic SODs, SodA and SodB. In addition, SodCI acts independently of the base excision repair system and RuvAB, involved in DNA repair. Although *sodCI* did show genetic interaction with *recA*, this was apparently independent of recombination and is presumably due to the pleiotropic effects of a *recA* mutation.

**Conclusions/Significance:**

Taken together, these results suggest that bacterial inhibition by phagocytic superoxide is primarily the result of damage to an extracytoplasmic target.

## Introduction

Macrophages normally kill bacteria by a coordinated delivery of toxic substances following phagocytosis. Phagosomes fuse with various membrane vesicles that deliver, for example, hydrolytic degradative enzymes and antimicrobial peptides. The NADPH-dependent oxidase (Phox), which produces superoxide, assembles in the phagosomal membrane [1]. Activated macrophages also produce nitric oxide, generated from arginine and oxygen by the inducible nitric oxide synthase (iNOS; [2]). Other reactive oxygen species (ROS) and reactive nitrogen species (RNS) can result [2], [3]. The phagocytic oxidative burst is a fundamental aspect of innate immunity, yet the mechanism by which these reactive species kill bacteria is not well understood.

*Salmonella enterica* serovar Typhimurium is a facultative intracellular pathogen that is capable of causing systemic infection in humans and mice by surviving within macrophages [4]–[6]. When engulfed by macrophages, serovar Typhimurium produces a series of virulence factors that allow the bacterium to inhibit the delivery of the antibacterial substances to the phagosome, to survive the various killing mechanisms, and to propagate in a unique compartment called the *Salmonella* containing vacuole [7]. Although *Salmonella* reduce delivery of Phox to the phagosome [8], [9], host production of ROS is clearly important for controlling infection [10], [11].

The NADPH-dependent oxidase complex in phagocytes generates superoxide from the univalent reduction of molecular oxygen [1]. At neutral pH, superoxide is charged and cannot penetrate membranes. However, the pKa of superoxide is approximately 4.8. Therefore, in the acidified phagosome, phagocytic superoxide could potentially be protonated, allowing flux into the bacterial cytoplasm [2], [12]. Superoxide is also produced endogenously in the bacteria by the inadvertent transfer of an electron to O_2_ from flavoproteins [13]. *E. coli* and *Salmonella* detoxify this endogenous superoxide using two cytoplasmic superoxide dismutases, SodA and SodB [3]. Cytoplasmic superoxide directly inactivates a set of dehydratases containing exposed [4Fe-4S] clusters and damages additional specific enzymes blocking several metabolic pathways [3]. Thus, mutants devoid of cytoplasmic superoxide dismutase (SOD) are auxotrophic for branched chain amino acids, sulfur-containing amino acids, and aromatic amino acids, and, due to defects in aconitase and fumarase, can grow only on fermentable carbon sources [14]. Damage to iron-sulfur clusters also causes the release of iron. Superoxide rapidly dismutes, either enzymatically or spontaneously, to form hydrogen peroxide (H_2_O_2_), which is reduced by the free iron to form hydroxyl radical (HO•) via the Fenton reaction. HO• is highly reactive and oxidation of biological molecules is diffusion limited. Because of the apparent propensity of positively charged iron to associate with the negatively charged DNA, H_2_O_2_ mediated cell death results from DNA damage [14]. Reduction of Fe^3+^ to Fe^2+^ by an unknown reductant allows the production of HO• to continue in the cell [14].

Many investigators have assumed that phagocytic superoxide kills by initiating DNA damage via the same mechanism described above for endogenously produced oxygen radicals [15]–[22]. We would argue that much of these data are based on experiments that fail to separate the effects of phagocytic and endogenously produced reactive oxygen species or other general defects in bacterial metabolism. For example, Buchmeier *et al.* [19] have previously shown that *recA* mutants of serovar Typhimurium are 3–4 logs less virulent than an isogenic wild type strain. The *recA* mutant was also sensitive to killing by tissue culture macrophages. It was presumed that this virulence defect is due to increased DNA damage in the *recA* mutant mediated by ROS produced by macrophages. However, *recA* mutants are generally defective and grow poorly compared to the wild type [23]. Thus, it is not clear whether this virulence defect is due to specific sensitivity to phagocytic ROS, or is a nonspecific effect caused by the altered growth rate of the mutant. Similarly, Suvarnapunya *et al.* [16] examined the effects of deleting components of the base excision repair system (BER) on *Salmonella* virulence. They showed that BER mutants were decreased in the ability to proliferate in tissue culture macrophages and were attenuated in mouse competition assays. Again, these results, per se, do not distinguish between a defect that is the direct result of phagocytic oxidative damage and oxidative damage mediated by endogenous sources of oxygen radicals.

The periplasmic copper-zinc cofactored superoxide dismutase, SodCI, is needed for full virulence of serovar Typhimurium [24]–[27]. Our strain of serovar Typhimurium also produces a second periplasmic SOD, SodCII, but this enzyme does not contribute to virulence [27]–[29]. Published data [30] and our data presented below indicate that SodCI specifically protects against phagocytic superoxide during infection. Based on this starting premise, and taking advantage of the genetic power of our system, we provide evidence that phagocytic superoxide primarily damages an extracytoplasmic target to inhibit or kill *Salmonella*. These results indicate that we need to reevaluate the dogma that DNA is a primary bacterial target of the phagocytic oxidative burst.

## Results

### SodCI protects specifically against phagocytic superoxide

The oxidative burst of phagocytes is a critical innate immune effector used to kill or inhibit invading bacteria, but the mechanism of action is unknown. The periplasmic superoxide dismutase, SodCI, specifically protects serovar Typhimurium against phagocytic superoxide. This conclusion is based on the following evidence. Complete deletion of the *sodCI* gene ([24], [31]; Tables 1 and 2) or point mutations that block SodCI enzymatic activity [31] attenuate virulence 8–17 fold in a competition assay after intraperitoneal infection. This attenuation is dependent on the production of phagocytic superoxide; in *phox−/−* knockout mice, a *sodCI* mutant had no phenotype (Table 2). This is consistent with previous results in cultured macrophages [30]. Moreover, in vitro, a strain deleted for both *sodCI* and *sodCII* shows no growth defect. The most sensitive measure of this is an *in vitro* competition assay, in which mutant and wild type are mixed one to one and grown overnight in a flask under highly aerated conditions. The *sodC* mutant and wild type strain competed evenly in this assay (Table 3). Thus, the *sodCI* mutant is specifically sensitive to phagocytic superoxide; there is neither a general growth defect nor apparent sensitivity to endogenous superoxide.

**Table 1:** Competition assays with SOD mutants in BALB/c mice.

Strain A	Strain B	Median CI	# of Mice	*p* a	Fold Attenuated^b^
*sodCI*	wt	0.13 †	21	<0.0005	8
*sodA*	wt	1.11 §	11	NS	*
*sodB*	wt	0.24 ‡	10	<0.0005	4
*sodA sodB*	wt	0.0013	9	<0.0005	770
*sodA sodB*	*sodB*	0.0029^c^	10	<0.0005	345
*sodCI sodA sodB*	*sodA sodB*	0.20^d^	5	<0.0005	5

a Student's t-test comparing CI versus inoculums.

NS or *, Not significant.

b Reciprocal of median CI.

c Significantly different (*p*<0.0005) versus §.

d Not significantly different (*p*>0.05) versus †.

Strains used: 14028, JS452, JS472, JS830-32.

**Table 2:** Competition assays in phox−/− and iNOS2 −/− mice.

Mouse Genotype	Strain A	Strain B	Median CI	# of Mice	*p* a	Fold Attenuated^b^
C57BL/6	*sodCI*	wt	0.18 ¶	21	<0.0005	5
C57BL/6 phox−/−	*sodCI*	wt	1.33^c^	5	NS	*
C57BL/6 iNOS2−/−	*sodCI*	wt	0.13^d^	6	<0.0005	8
C57BL/6	*sodAB*	wt	0.0018	6	<0.0005	556
C57BL/6 phox−/−	*sodAB*	wt	0.012	6	<0.0005	83

a Student's t-test comparing CI versus inoculums.

NS or *, Not significant.

b Reciprocal of median CI.

c Significantly different (*p*<0.0005) versus ¶.

d Not significantly different (*p*>0.05) versus ¶.

Strain used: 14028, JS472.

**Table 3:** Competition assays in aerated LB cultures.

Strain A	Strain B	Median CI	# Samples	*p* a	Fold Attenuated^b^
*sodCI sodCII*	wt	0.96	5	NS	*
*sodA sodB*	wt	0.0002	5	<0.0005	5000
*xthA nfo*	wt	0.49	5	0.0047	2
*ruvAB*	wt	0.10	5	<0.0005	10
*recA*	wt	0.016	5	<0.0005	62
*sodCI recA*	*recA*	0.85	5	NS	*

a Student's t-test comparing CI versus inoculums.

NS or *, Not significant.

b Reciprocal of median CI.

Strains used: 14028, JS456, JS831, JS835, JS838, JS843, JS845.

The defect conferred in the animal by loss of SodCI is the direct result of superoxide. Superoxide spontaneously dismutes to hydrogen peroxide, which can subsequently be converted into additional downstream reactive oxygen species. SodCI simply enhances the rate of dismutation and lowers the steady state concentration of superoxide. Thus, equal amounts of hydrogen peroxide and downstream reactive oxygen species are expected whether SodCI is present or not. Since superoxide and nitric oxide can react to form peroxynitrite, it is possible that the protective role of SodCI in the host is to prevent the formation of this highly reactive antimicrobial substance. To test this, we performed a competition assay in iNOS deficient mice. The data show that the *sodCI* mutant was 8-fold attenuated in this background compared to the wild type strain and this defect was not significantly different than that observed in the parent C57BL/6 mice (Table 2). Since the *sodCI* mutant still shows a virulence defect in iNOS mice, the role of SodCI in the host is apparently not to protect against peroxynitrite. This is consistent with published data suggesting a temporal separation between the effects of superoxide and nitric oxide in protecting against *Salmonella* infection [11]. Taken together, these data show that SodCI directly protects the bacterial cell against phagocytic superoxide.

### SodCI acts independently of cytoplasmic SODs

Since the only biological molecules in bacteria known to be damaged by superoxide are located in the cytoplasm, we tested the hypothesis that SodCI protects a cytoplasmic target. In this series of experiments, we take advantage of the genetic concept of “synthetic phenotypes” [32]. If two gene products contribute independently to the same process, then the combination of the two mutations should give a phenotype that is more severe than what would be predicted by the simple combination of the two individual mutations. The competition assay is amenable to this type of genetic analysis [33], [34]. Following this rationale, we conducted a series of competitive virulence assays involving *sodCI* mutants.

It is established that SodA and SodB prevent DNA damage [35] and inactivation of sensitive enzymes [3], [14] caused by superoxide in the cytoplasm. We tested the effects of deleting *sodA* and *sodB* in the mouse competition assay. As reported previously [36], deletion of *sodA* did not significantly affect virulence (Table 1). In contrast, deletion of *sodB* conferred a mild but significant virulence defect; 4 fold in a competition assay (Table 1). These data suggest that SodB contributes the majority of the cytoplasmic superoxide dismutase activity during growth of *Salmonella* in the animal. We then tested the effect of deleting *sodA* in the *sodB* background by directly competing a *sodA sodB* double mutant against the *sodB* single mutant. The *sodA sodB* double mutant was highly attenuated (Table 1). This is an example of a synthetic phenotype and provides proof of principle for both our rationale and the use of the animal competition assay for this analysis. Because SodA and SodB both protect the cytoplasm from damage by superoxide, in the absence of SodB, the further loss of SodA has a dramatic effect, 300-fold attenuation, whereas in the wild-type background, loss of SodA has a negligible effect on virulence. As striking as this phenotype is, we cannot simply ascribe the virulence defect to phagocyte derived superoxide. Indeed, in an in vitro competition assay in LB broth, the *sodA sodB* mutant was highly compromised in its ability to grow aerobically compared to the wild-type (Table 3). Moreover, the *sodA sodB* mutant remained attenuated in *phox−/−* knockout mice (Table 2). In other words, phagocytic superoxide is not required to “attenuate” the *sodA sodB* double mutant.

If the role of SodCI is to protect the cytoplasm from phagocytic superoxide, then a *sodCI* mutation should be synthetic with mutations in *sodA* and *sodB*. This was not observed. In the *sodA sodB* background, a *sodCI* mutation was 5-fold attenuated, which was not significantly different than the 8-fold attenuation observed in the wild type background (Table 1). Thus, SodCI acts independently of the cytoplasmic superoxide dismutases. This strongly suggests that SodCI protects an extracytoplasmic target.

### SodCI acts independently of base excision repair

Because superoxide can potentiate DNA damage, we further examined the possibility that SodCI protects DNA from phagocyte derived superoxide. The base excision repair system (BER) is critical for the repair of DNA damage mediated by ROS [37]. The *xthA* gene product, ExoIII, and the *nfo* gene product, EndoIV, remove oxidatively damaged bases [38] or other fragments left after oxidative DNA damage, or the deoxyribose moieties that result from removal of damaged bases by other N-glycosylases [39]. An *xthA nfo* double deletion mutant was constructed via lambda Red-mediated recombination [40]. To further confirm that these mutations had the appropriate phenotype, we showed that the *xthA nfo* mutations were synthetically lethal with *recA* ([41];data not shown).

The *xthA*/*nfo* mutant was 4-fold attenuated in an i.p. competition assay (Table 4). This result is consistent with the data of Suvarnapunya *et al.* [16]. However, the *xthA/nfo* mutant was 2-fold attenuated in an in vitro competition assay in LB broth (Table 3), and, therefore, the attenuation does not prove that phagocytic oxygen radicals mediate this apparent DNA damage. We then tested the phenotype conferred by the *sodCI* deletion in an *xthA*/*nfo* background. The result was not significantly different than the phenotype conferred by deletion of *sodCI* in the wild type background (Table 4). As a control for this experiment, we tested the phenotype conferred by the *sodB* deletion in the *xthA/nfo* background. As expected, the combination of these mutations conferred a synthetic phenotype. The *sodB* mutation conferred 15-fold attenuation in the *xthA/nfo* background (Table 4). In *E. coli*, *sodA*/*sodB* and *xthA/nfo* are known to be synthetic in vitro [35]. Genetically, these results show that XthA, Nfo, and SodB participate in the same process, protecting the DNA from oxidative damage. In contrast, SodCI acts independently of XthA/Nfo, strongly arguing that attenuation in the *sodCI* mutant is not the result of oxidative DNA damage.

**Table 4:** Competition assays with BER mutants in BALB/c mice.

Strain A	Strain B	Median CI	# Mice	*p* a	Fold Attenuated^b^
*xthA nfo*	wt	0.24	9	0.0065	4
*sodCI xthA nfo*	*xthA nfo*	0.16^c^	4	0.0006	6
*sodB xthA nfo*	*xthA nfo*	0.065^d^	9	<0.0005	15

a Student's t-test comparing CI versus inoculums.

NS or *, Not significant.

b Reciprocal of median CI.

c Not significantly different (*p*>0.05) versus †, Table 1.

d Significantly different (*p*<0.03) versus ‡, Table 1.

Strains used: 14028, JS835-37.

### SodCI acts independently of recombinational repair but is affected by mutations in *recA*

Double strand breaks are another possible form of damage caused indirectly by superoxide via hydroxyl radical formation [35]. This type of lesion is repaired by the *recBCD* recombination pathway, which requires RecA, RecBCD, and the helicase RecG or the RuvABC resolvasome [23]. Buchmeier et al. reported that *recA* and *recBC* mutants were attenuated in mice and exhibited decreased survival in the J774.16 macrophage cell line [19]. This defect was partially suppressed in the D9 variant of macrophage cell line J774.16, which is deficient for NADPH oxidase. Since this suggested that recombinational repair may be necessary to protect DNA from phagocytic superoxide, we asked genetically if SodCI participates with this repair pathway to protect DNA. In the mouse competition assay, the *recA* deletion mutant was 56-fold attenuated (Table 5). Again, this defect cannot simply be attributed to phagocytic ROS effects; the *recA* strain was 62-fold decreased in competitive index *in vitro* (Table 3). As expected, a *sodB recA* double mutant showed a synthetic phenotype in the mouse competition assay; deletion of *sodB* conferred 244-fold attenuation in the *recA* background compared to 4-fold in a wild type background (Table 5). This is also consistent with in vitro results with *E. coli*, where *sodA*/*sodB* and *recA* are known to be synthetic [35]. We then tested the effect of deleting *sodCI* in a *recA* background. Surprisingly, *sodCI* showed a slight synthetic interaction with *recA*, 32-fold attenuated compared to 8-fold attenuated in the wild type background (Table 5). Although much less dramatic than the synthetic interaction between *sodB* and *recA*, this result was reproducible and statistically significant. This synthetic phenotype is dependent on phagocytic superoxide; loss of SodCI showed no phenotype in vitro in a *recA* background (Table 3).

**Table 5:** Competition assays with recombination deficient strains in BALB/c mice.

Strain A	Strain B	Median CI	# of Mice	*p* a	Fold Attenuated^b^
*recA*	wt	0.019	13	<0.0005	56
*sodB recA*	*recA*	0.0041^c^	3	<0.0005	244
*sodCI recA*	*recA*	0.031^d^	12	<0.0005	32
*ruvAB*	wt	0.0064	4	<0.0005	156
*sodB ruvAB*	*ruvAB*	0.028^e^	5	0.0005	36
*sodCI ruvAB*	*ruvAB*	0.19^f^	9	<0.0005	5
*lexA3*	*wt*	1.42	4	NS	*
*sodCI lexA3*	*lexA3*	0.22^f^	4	0.0029	5

a Student's t-test comparing CI versus inoculums.

NS or *, Not significant.

b Reciprocal of median CI.

c Significantly different (*p* = 0.001) versus ‡, Table 1.

d Significantly different (*p* = 0.0008) versus †, Table 1.

e Significantly different (*p* = 0.05) versus ‡, Table 1.

f Not significantly different (*p*>0.05) versus †, Table 1.

Strains used: 14028, JS838-45.

Given the apparent synthetic interaction between *sodCI* and *recA*, we tested other members of the recombinational repair pathway. Deletion of *recBCD* attenuated *Salmonella* in the mouse competition assay to such a level that it was difficult to accurately measure additional defects and we could draw no conclusions from double mutants (data not shown). We then asked genetically if SodCI participates with RuvAB to protect DNA. In i.p. competition assays the *ruvAB* mutant was 150-fold attenuated compared to the wild-type strain (Table 5). This mutant was also 10-fold attenuated when competed against the wild type strain in vitro (Table 3). As a control, we examined the effect of loss of SodB in the *ruvAB* background. As expected, *sodB* was synthetic with *ruvAB*, conferring a 36-fold defect in this background (Table 5). We then tested the phenotype of a *sodCI* mutant in the *ruvAB* background. In this case *sodCI* was 5-fold attenuated in the *ruvAB* background, indicating that SodCI acts independently of RuvAB (Table 5). Thus, in contrast to the results obtained above, these results suggest that SodCI acts independently of recombinational repair.

Taken together, the data above suggest that the synthetic phenotype observed in the *recA sodCI* double mutant is not a result of the defect in recombinational repair. The RecA protein also plays a regulatory role in the cell, indirectly controlling gene expression, mainly via LexA and the SOS response. It was possible that the synthetic phenotype was the result of the lack of SOS induction rather than the loss of recombinational repair. Therefore, we tested the role of SOS in resistance to phagocytic superoxide. We introduced a non-inducible *lexA* allele (*lexA3*; [42]) into serovar Typhimurium. To confirm the presence and phenotype of this allele, we created an *sulA* ^+^-*lac* ^+^ transcriptional fusion [43] and transduced this fusion construct into isogenic wild type and *lexA3* strains. As a control, we transduced the Δ*recA*::Tc allele into the wild type background. As shown in Table 6, the *sulA* fusion in the wild type background was highly induced in response to nalidixic acid treatment, whereas, as expected, neither the *recA* or *lexA3* strains were capable of inducing the SOS-dependent fusion.

**Table 6:** Confirmation of *lexA3* uninducible allele.

Strain^a^	β-gal Activity^b^	
	−Nal	+Nal
WT	51.3±0.74	1491.8±30
*recA*	47.3±0.23	20.7±0.65
*lexA3*	44.2±0.83	31.2±1.6

a Strains (JS846-848) contain a *sulA^+^-lac^+^* transcriptional fusion.

b Mid-log LB cultures were split and 10 µg/ml nalidixic acid (Nal) was added to one half. β-galactosidase activity was determined after 3 hrs further incubation. Units are defined as (µmol of ONP formed min^−1^)×10^6^/(OD600×ml of cell suspension) and are reported as mean±standard deviation where n = 4.

The *lexA3* strain was fully virulent in a competition assay (Table 5). These data suggest that *Salmonella* is not overcoming significant DNA damage in order to survive in the host. If the synthetic phenotype observed between *sodCI* and *recA* in the animal was due to the inability of the *recA* strain to induce SOS, then *sodCI* should also show a synthetic phenotype in the *lexA* non-inducible strains. This was not observed. As shown in Table 5, *sodCI* conferred its normal phenotype in the *lexA3* background. Thus, the increased sensitivity to phagocytic superoxide observed in a *recA* mutant is apparently independent of both SOS induction and recombinational repair.

### Sensitivity to in vitro generated superoxide and hydrogen peroxide

**Figure 1:**
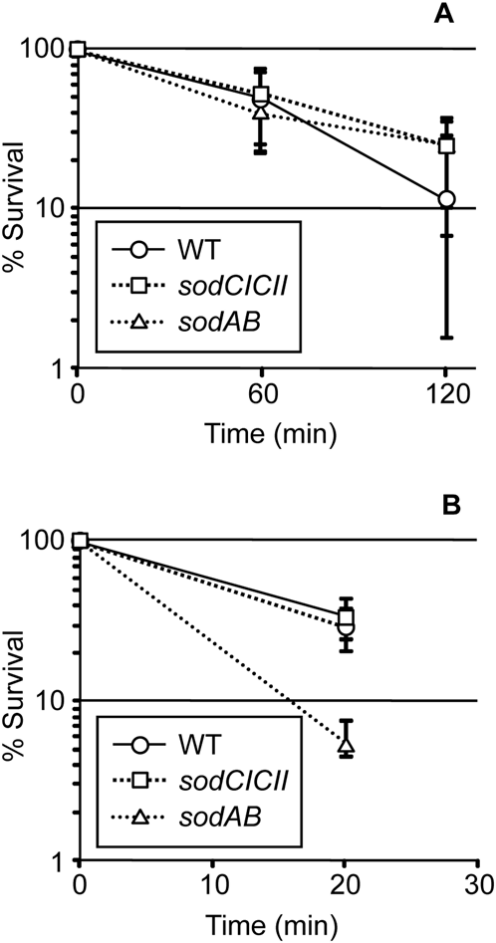
Effect of *sod* mutations on survival in cells exposed to exogenous reactive oxygen species. A) From triplicate cultures, 0.25 mM hypoxanthine with or without 0.1 u/ml xanthine oxidase was added to ∼10^6^ cfu/ml of cells in PBS. Colony forming units were determined at the indicated time points. The cfu of untreated samples remained essentially constant throughout the experiment. The viable count of the treated sample is compared to untreated sample at each time point. B) Triplicate mid-log cultures in LB 0.2% glucose were split and 2.5 mM H_2_O_2_ was added to one half. Colony forming units were determined and compared to untreated sample at each time point as above. The cfu of untreated samples increased only slightly during the experiment. The mean and range are plotted for each experiment. Strains used: 14028, JS456, JS831.

The data above suggest that the most vulnerable target of phagocytic superoxide is extracytoplasmic. But identifying the target(s) is complicated by the fact that *sodC* mutants of *Salmonella* show no significant in vitro phenotypes. As shown in Figure 1A, there was no significant difference in sensitivity to 250 µM hypoxanthine/xanthine oxidase of the wild type and isogenic mutants devoid of periplasmic SODs or cytoplasmic SODs. We have performed similar experiments using this and other superoxide-generating systems under a variety of conditions and have never observed a reproducible difference between the wild type and *sodCI sodCII* double mutant. This should not be surprising. It is estimated that NADPH oxidase is capable of producing a steady state superoxide concentration of 100 µM in a phagosome [12]. Using published data [44], [45], one can calculate that in vitro systems such as that used above are capable of generating only <1 µM of superoxide for a few minutes. When higher concentrations (2.5 mM) of hydrogen peroxide were used, the *sodA sodB* double mutant showed increased sensitivity (Figure 1B), as previously reported [46]. The *sodCI sodCII* mutant behaved identically to wild type, again distinguishing loss of cytoplasmic versus periplasmic SOD activity.

## Discussion

The phagocytic NADPH oxidase plays a central role in the antimicrobial arsenal of the innate immune response. This is evidenced by the increased susceptibility of both humans and mice that lack the NADPH oxidase to a variety of bacterial infections, including *Salmonella* [10], [11], [47]. The periplasmic superoxide dismutase SodCI in *Salmonella* specifically protects the bacterium from phagocytic superoxide. Starting with this premise, we tested the common assumption that the primary target of the phagocytic oxidative burst is the bacterial DNA or other cytoplasmic target. Our results show that SodCI acts independently of SodA and SodB, strongly suggesting that phagocytic superoxide is not gaining access to the cytoplasm. Furthermore, we could exclude DNA as a target of phagocytic superoxide based on the lack of genetic interaction between *sodCI* and genes encoding base excision repair (*xthA nfo*) or recombinational DNA repair, *ruvAB*. These data provide evidence that phagocytic superoxide specifically damages an extracytoplasmic target to inhibit or kill *Salmonella*. The fact that *sodCI* mutants are significantly attenuated despite the prowess of *Salmonella* to survive in macrophages suggests that extracytoplasmic damage by superoxide is the primary vulnerability to the oxidative burst in macrophages and there is no reason to think that our results do not apply in general to macrophage bacterial killing. To our knowledge, this is the first evidence that such an extracytoplasmic target exists, although it could be inferred by the periplasmic localization of the superoxide dismutase.

SodCI is protecting against direct damage by superoxide, given that the periplasmic superoxide dismutase will decrease the steady state concentration of superoxide but not ultimately change the yield of hydrogen peroxide or other downstream reactive oxygen species. Our results do not address the potential damage to cytoplasmic targets, including DNA, caused by these downstream reactive oxygen species. However, in vivo evidence for such damage is surprisingly limited, and our results suggest that simply showing that mutants that are sensitive to cytoplasmic oxidative stress are attenuated in the host is not necessarily meaningful. Indeed, several studies suggest that the cytoplasm is not under increased oxidative stress during infection. Serovar Typhimurium strains mutant in *soxS* [48] or *oxyR* [49], whose products regulate adaptation to cytoplasmic oxidative stress [3], are fully virulent in an animal model, as are mutants incapable of inducing LexA-dependent SOS (Table 5). Strains lacking catalase (*katE katG*; [20]) or alkyl hydroperoxide reductase (*ahpCF*; [49]) are also fully virulent, suggesting that there is enough redundancy in these peroxide scavenging systems to the keep the cytoplasmic H_2_O_2_ level below 5 µM during growth in the host [50]. Schloss-Silverman *et al.* measured mutant frequency and plasmid nicking in *E. coli* and serovar Typhimurium recovered from J774 macrophage line. *E. coli* showed increased DNA damage, but serovar Typhimurium did not [51]. Moreover, it is well established that cytoplasmic superoxide or hydrogen peroxide damage key enzymes in metabolic pathways at concentrations below that required to cause significant DNA damage [14], leading to, for example, aromatic amino acid auxotrophy and blocks in the TCA cycle, both of which are known to significantly attenuate *Salmonella* [52], [53]. Taken together, these results suggest that those *Salmonella* cells that survive in the host are not experiencing cytoplasmic oxidative stress or significant oxidative DNA damage.

Our results seem to exclude any cytoplasmic damage from phagocytic superoxide, yet, in contrast to the results with base excision repair and *ruvAB*, we observed a genetic interaction between periplasmic superoxide dismutase and RecA. It must be noted that RecA has multiple roles in the cell in addition to its direct action in recombination. Most importantly, RecA controls the expression of more than 40 genes. Most of these are under the control of the LexA repressor, but we could show that the genetic interaction with SodCI is not dependent on the LexA-dependent SOS response. However, RecA is also known to regulate a number of genes/proteins independently of LexA. These include: 2-keto-4-hydroxygluterate aldolase, required for recovery of a respiratory block induced by UV irradiation [54]; DinY [55]; and the universal stress proteins UspA, UspC, UspD, and UspE [56], which have multiple roles in the cell, including effects on extracytoplasmic processes [57]. Lesca et al. [58] noted 14 proteins by two-dimensional gel electrophoresis that were induced in a RecA-dependent fashion but independent of LexA. The simplest explanation of our results is that in the *recA* mutant pleiotropic effects result in increased sensitivity to extracytoplasmic superoxide.

Segal and colleagues have proposed that, in neutrophils, the role of superoxide production by the phagocyte oxidase is not to kill bacteria per se, but rather to deliver electrons into the phagosome. This stimulates an influx of K^+^ and a rise in the pH to ∼7.5, which are required for the activity of granule proteases, proposed to be the ultimate cause of bacterial death [59], [60]. One could invoke similar arguments to explain our results, but they do not withstand criticism. *Salmonella* grows in macrophages rather than neutrophils [5], [6] and the overall killing mechanisms differ between the two phagocytic cell types [61]. More importantly, if the sole function of producing superoxide is to introduce electrons into the phagosome, then the periplasmic superoxide dismutase, SodCI, should be irrelevant. Dismutation of superoxide to H_2_O_2_ does not change the electron flux.

We currently do not know what the extracytoplasmic target of superoxide could be. The known biological molecules damaged directly by superoxide are quite limited and include certain dehydratases containing solvent exposed iron sulfur clusters [14]. No enzymes of this class are known to be localized to the periplasm. The Tat system is responsible for secretion of a series of extracytoplasmic proteins with cofactor-dependent reaction centers [62] that are potential targets of extracellular superoxide, but we have shown that Tat and SodCI are genetically independent (data not shown). Superoxide does not damage proteins per se, nor is it expected to react with bacterial membranes [14]. These studies are complicated by the fact that the only significant and reproducible phenotype of a *sodCI* mutant is in the animal infection model. Other investigators have reported modest (ca. 2 fold) differences in the rate of killing of various *sodC* mutants in the presence of (hypo)xanthine/xanthine oxidase [25], [30], [63], [64]. Control experiments showed this sensitivity to be hydrogen-peroxide dependent [63]. However, we observe no such sensitivity in our background under a variety of in vitro conditions. Identification of the target will have to await further investigation.

## Materials and Methods

### Ethics Statement

All animal work was reviewed and approved by the University of Illinois IACUC and performed under protocols 04137 and 07070.

### Bacterial Strains and Growth Conditions

Bacterial strains and plasmids are described in Table 7. All *Salmonella enterica* serovar Typhimurium strains used in this study are isogenic derivatives of strain 14028 (American Type Culture Collection) and were constructed using P22 HT105/1 *int*-201 (P22) mediated transduction [65]. Deletion of various genes and concomitant insertion of an antibiotic resistance cassette was carried out using lambda Red-mediated recombination [40] as described in [43]. Primers were purchased from IDT Inc. The endpoints of each deletion are indicated in Table 7. In all cases, the appropriate insertion of the antibiotic resistance marker was checked by PCR analysis. The constructs resulting from this procedure were moved into a clean wild type background (14028) by P22 transduction. Antibiotic resistance cassettes were removed using the temperature sensitive plasmid pCP20 carrying the FLP recombinase [66].

**Table 7:** Bacterial strains used in this study.

Strain	Genotype^a^	Deletion End Points^b^	Source or Reference^c^
14028	Wild type serovar Typhimurium		ATCC^d^
JS452	Δ*sodB102*::Km	1509486–1509923	[27]
JS454	Δ*sodCII-103*::Cm	1516106–1516488	[27]
JS456	Δ*sodCI-1*::*aph* Δ*sodCII-103*::Cm		[27]
JS472	Δ*sodCI-1*::*aph*		[27]
JS830	Δ*sodA112*::Cm	4266594–4266789	
JS831	Δ*sodA112*::Cm Δ*sodB102*::Km		
JS832	Δ*sodA112*::Cm Δ*sodB102* Δ*sodCI-1*::*aph*		
JS833	Δ*xthA51*::Cm	1380972–1381714	
JS834	Δ*nfo1*::Km	2302687–2303476	
JS835	Δ*xthA51*::Cm Δ*nfo1*::Km		
JS836	Δ*xthA51*::Cm Δ*nfo1* Δ*sodB102*::Km		
JS837	Δ*xthA51*::Cm Δ*nfo1* Δ*sodCI-1*::*aph*		
JS838	Δ*ruvAB*::Cm	1989088–1990664	
JS839	Δ*ruvAB*::Cm Δ*sodCI-1*::*aph*		
JS840	Δ*ruvAB*::Cm Δ*sodB102*::Km		
JS841	Δ*lexA33*::[Cm *lexA3*(Ind^−^)](sw)		
JS842	Δ*lexA33*::[Cm *lexA3*(Ind^−^)](sw) Δ*sodCI-1*::*aph*		
JS843	Δ*recA711*::Tc	2974870–2975903	
JS844	Δ*recA711*::Tc Δ*sodB102*::Km		
JS845	Δ*recA711*::Tc Δ*sodCI-1*::*aph*		
JS846	Φ(*sulA^+^-lac^+^*)*111*		
JS847	Φ(*sulA^+^-lac^+^*)*111* Δ*recA711*::Tc		
JS848	Φ(*sulA^+^-lac^+^*)*111* Δ*lexA33*::[Cam *lexA3*(Ind^−^)](sw)		

a All strains are isogenic derivatives of 14028.

b Numbers indicate the base pairs that are deleted or cloned (inclusive) as defined in the *S. enterica* serovar Typhimurium LT2 genome sequence in the National Center for Biotechnology Information Database.

c This study, unless otherwise noted.

d ATCC, American Type Culture Collection.

Luria-Bertani (LB) medium was used in all experiments for growth of bacteria. Bacterial strains were routinely grown at 37°C except for strains containing the temperature sensitive plasmids, pCP20 or pKD46 [40], which were grown at 30°C. Antibiotics were used at the following concentrations: 20 µg/ml chloramphenicol; 50 µg/ml kanamycin; and 12 µg/ml tetracycline.

Bunny et al. [42] recombined the uninducible *lexA3* allele from *E. coli* into the *Salmonella* Typhimurium genome linked to a Cm marker. We moved this allele into our strain background via P22 HT105/1 *int*-201 (P22) mediated transduction [65]. LB cultures of each strain were grown to mid-log phase and nalidixic acid was added to a final concentration of 10 µg/ml. After 3 hours incubation at 37°C, the β-galactosidase activity produced from the fusion in each strain was measured using a microtiter plate assay as previously described [67]. β-galactosidase activity units are defined as (µmol of ONP formed min^−1^)×10^6^/(OD600×ml of cell suspension) and are reported as mean±standard deviation where n = 4.

### Competition Assays

BALB/c mice (BALB/cAnNHsd) were purchased from Harlan Sprague Dawley, Inc. C57BL/6 and congenic phox−/− (B6.129S6-*Cybb^tm1Din^*/J) and iNOS2−/− (B6.129P2-*Nos2^tm1Lau^*/J) mice were from Jax Mice. Bacterial strains were grown overnight (16 h) in LB medium. Cultures of the two strains of interest were mixed 1∶1 and the mixture was washed, and diluted in sterile 0.15 M NaCl. For competition assays, 6–8 week old female mice were inoculated intraperitoneally (i.p.) in groups of 4 to 6 with the mixture of the two bacterial strains (approximately 500 total bacteria). Inocula were plated on LB and then replica plated onto the appropriate selective media to determine the total number and percentage of the two strains used for the infection. Mice were sacrificed after 4 to 5 days of infection and their spleens were removed. The spleens were homogenized, diluted and plated on LB medium. The resulting colonies were replica plated onto the selective medium to determine the relative percent of each strain recovered. The competitive index (CI) was calculated as follows: (percent strain A recovered/percent strain B recovered)/(percent strain A inoculated/percent strain B inoculated). The CI of each set of assays was analyzed statistically using the Student's t test. In most cases, the strains were rebuilt by P22 transduction, and the mouse assay was repeated to ensure that the virulence phenotypes were the result of the designated mutations.

Growth and dilution of bacteria for *in vitro* competitions were performed as above, but 0.1 ml of the bacterial mixture was introduced into 5 ml of LB in a 50 ml flask. Flasks were incubated at 37° on a platform shaker rotating at 225 RPM for 18 hrs. Cells were diluted and plated on LB medium, and the resulting colonies were replica plated onto the selective medium to determine the relative percent of each strain recovered. Competitive index was calculated as above and the Student's t-test was used for statistical analyses.

### Sensitivity to external superoxide and H_2_O_2_ in vitro

To test sensitivity to external superoxide, triplicate cultures grown overnight in LB medium were diluted 1/100 in LB and grown in highly aerated flasks to OD_600_ of 0.2. Cells were washed and diluted to approximately 10^6^ cfu/ml in phosphate-buffered saline (PBS) pH 7.4 containing 250 µM hypoxanthine. Samples were split and either remained untreated or xanthine oxidase (Sigma) was added to 0.1 unit/ml. The samples were incubated at 37°C with aeration. At the given time points, cells were diluted in sterile 150 mM NaCl and plated for colony forming units. Samples with xanthine oxidase were compared to untreated samples at each time point.

Strains were assessed for H_2_O_2_ sensitivity as previously described [68]. Triplicate overnight LB cultures were diluted in LB medium with 0.2% glucose to OD_600_ of 0.01 and grown in highly aerated flasks to OD_600_ of 0.2. Cells were diluted 1/100 in LB with 0.2% glucose, split, and one part was exposed to 2.5 mM H_2_O_2_ for 20 min at 37°C. Samples were immediately diluted in sterile 150 mM NaCl and plated for colony forming units. Samples with H_2_O_2_ were compared to untreated samples.
